# Application of Machine Learning to the Prediction of Surface Roughness in the Milling Process on the Basis of Sensor Signals

**DOI:** 10.3390/ma18010148

**Published:** 2025-01-02

**Authors:** Katarzyna Antosz, Edward Kozłowski, Jarosław Sęp, Sławomir Prucnal

**Affiliations:** 1 Faculty of Mechanical Engineering and Aeronautics, Rzeszow University of Technology, 35-959 Rzeszow, Poland; jsztmiop@prz.edu.pl (J.S.); spktmiop@prz.edu.pl (S.P.); 2 Faculty of Management, Lublin University of Technology, 20-618 Lublin, Poland; e.kozlovski@pollub.pl

**Keywords:** roughness, machining, milling process, machine learning, elastic net, neural network, Savitzky–Golay filter

## Abstract

This article presents an investigation of the use of machine learning methodologies for the prediction of surface roughness in milling operations, using sensor data as the primary source of information. The sensors, which included current transformers, a microphone, and displacement sensors, captured comprehensive machining signals at a frequency of 10 kHz. The signals were subjected to preprocessing using the Savitzky–Golay filter, with the objective of isolating relevant moments of active material machining and reducing noise. Two machine learning models, namely Elastic Net and neural networks, were employed for the prediction purposes. The Elastic Net model demonstrated effective handling of multicollinearity and reduction in the data dimensionality, while the neural networks, utilizing the ReLU activation function, exhibited the capacity to capture complex, nonlinear patterns. The models were evaluated using the coefficient of determination (R²), which yielded values of 0.94 for Elastic Net and 0.95 for neural networks, indicating a high degree of predictive accuracy. These findings illustrate the potential of machine learning to optimize manufacturing processes by facilitating precise predictions of surface roughness. Elastic Net demonstrated its utility in reducing dimensionality and selecting features, while neural networks proved effective in modeling complex data. Consequently, these methods exemplify the efficacy of integrating data-driven approaches with robust predictive models to improve the quality and efficiency of surface.

## 1. Introduction

Due to its versatility, precision, and ability to produce complex geometries, milling plays a vital role in machining processes across a wide range of industries. It accounts for approximately 38% of all machining processes, surpassing turning (34%) and drilling (23%), with other processes, such as grooving and parting, making up the remaining 5% [1]. In the milling machine market, vertical milling machines dominate with a 52.6% share, followed by horizontal milling machines with 38.9%, underlining the importance of milling machines [2,3]. The importance of milling is particularly evident in industries such as automotive, aerospace, and general manufacturing, where it accounts for 30–50% of machining operations, depending on the application [2,4]. Examples include the production of turbine blades, engine components, and molds that are critical to the aerospace and defense sectors [3]. The global CNC (Computerized Numerical Control) milling machine market, valued at $82.48 billion in 2023, is forecast to grow at a CAGR (Compound Annual Growth Rate) of 3.92% to reach $172.31 billion by 2032, highlighting its continued importance and market expansion [2].

The milling process has a significant effect on the roughness of the surface of the materials, and several factors influence the final surface quality. Key parameters such as cutting speed, feed rate, and depth of cut play a crucial role in determining surface roughness. For example, studies have shown that lower values of these parameters tend to increase surface roughness, as observed in experiments on mild steel using the Taguchi method and ANOVA analysis [5]. Similarly, when SiCp/Al materials were milled, an increase in the feed rate and the milling speed was found to increase the surface roughness, with the milling speed being the dominant factor that affects the microstructure of the machined surface [6]. The influence of spindle speed is also highlighted, and higher speeds generally result in smoother surfaces, as demonstrated in the end milling of aluminum alloys [7]. In addition, the presence of machining vibrations and tool trajectories can further complicate the finish of the surface, requiring predictive models that incorporate these variables to maintain the desired surface quality [8]. The use of advanced modeling techniques such as SSA-LSSVM and the response surface methodology has been effective in predicting and evaluating surface roughness with high precision, taking into account the uncertainty and coupling effects of various factors [9]. In addition, the cutting force during milling, which affects elastic–plastic deformation, is another critical factor, with increased cutting force generally reducing surface roughness under constant cutting parameters [10]. Optimization of these parameters, as demonstrated in the grinding of SKD11 steel using the Taguchi method, can lead to improved surface conditions, highlighting the importance of precise parameter control to achieve optimal surface roughness [11].

In general, understanding and controlling these factors is essential to improve the sustainability of machining and reduce the need for additional finishing processes [12].

Assessment of surface roughness after grinding is a multifaceted topic that involves different methods and parameters that influence the final surface quality. Several studies have explored different approaches to predict and analyze surface roughness, emphasizing the importance of cutting parameters such as speed, feed, and depth of cut. For example, Shailesh et al. used the Taguchi method and ANOVA to determine that lower values of these parameters generally result in higher surface roughness when milling mild steel under wet conditions [5]. Similarly, Daniyan et al. used the Response Surface Methodology (RSM) to develop a predictive model for AISI D3 alloy steel, highlighting the significant influence of feed rate, depth of cut, and cutting speed on surface roughness [13].

Advanced predictive models using machine learning (ML) techniques have been explored through several innovative approaches to improve prediction accuracy. In addition to these computational methods, experimental techniques such as atomic force microscopy (AFM), which can isolate surface features with sub-nanometer resolution and subtract background topology contributions [14], and laser-based profilometers, which offer high accuracy and real-time roughness measurements [15], are widely used. While these tools are effective for detailed analysis, machine learning methods offer advantages such as reduced data acquisition time, the ability to generalize across varying conditions, and the potential for real-time integration into manufacturing processes. This positions ML as a complementary and scalable solution for roughness assessment in industry.

Lai et al. introduced a system that uses regression analysis and neural networks to predict surface roughness by integrating real-time data from CNC machines [16]. Tsai et al. applied various neural network models, including CNN (Convolutional Neural Network) and LSTM (Long Short-Term Memory), to predict surface roughness in SUS304 stainless steel and found that CNN provided the best analytical efficiency [17]. In addition, Wang et al. proposed an NCA-SAE(Neighborhood Component Analysis (NCA) and Sparse Autoencoder (SAE)) feature fusion method optimized by particle swarm optimization to improve prediction accuracy [18].

The use of fuzzy logic by Amira et al. also demonstrated high precision in predicting surface roughness for Ti-6Al-4V, emphasizing the role of cutting parameters [19]. In addition, Shang et al. developed a virtual metrology model using a backpropagation neural network that achieved high prediction accuracy by incorporating multiple sensor data inputs [20]. Taken together, these studies underscore the complexity of surface roughness assessment in milling, highlighting the interaction of process parameters and the potential of advanced modeling techniques to improve surface quality prediction and control.

Deep transfer learning models, such as Deep CORAL AlexNet, have been used to detect surface roughness levels using color image data, achieving a cross-domain detection accuracy of 99.39% even under complex lighting conditions, demonstrating robustness and a wide measurement range [21]. Support vector machines (SVMs) have also been used to predict surface roughness in end milling operations, with error margins between 0.4% and 10%, although the correlation with current signals was less effective due to variations in tool wear [22]. Neural networks, particularly feedforward backpropagation neural networks (BPNNs), have shown high prediction accuracy, with BPNNs achieving 99.85% accuracy in predicting surface roughness in eco-friendly milling processes [23]. Convolutional neural networks (CNNs) and long-short-term memory (LSTM) networks have been applied to predict surface roughness and machining accuracy, with CNNs showing superior analytical efficiency [14]. Deep learning methods based on physics have been proposed to integrate physical laws into ML models, improving prediction accuracy by reducing the mean absolute percentage error by 3.03% compared to traditional methods [23]. In addition, neural networks of the radial basis function and fuzzy modeling have been used to predict surface roughness in MDF milling, and fuzzy logic achieves a lower estimation error [24].

The integration of multiple sensor data, such as force, vibration, and current, into a back-propagation neural network has also been shown to improve prediction accuracy, achieving a mean absolute percentage error of 1.01% [18]. Broad learning systems have been proposed to overcome the limitations of deep learning models, offering fast training speeds and high detection accuracy for roughness classification [25,26,27,28,29].

Machine vision integrated with ML has been used to predict surface roughness from images, using grayscale intensity patterns to develop and validate predictive models [30].

Nguyen et al. demonstrated that different machine learning models, including Extreme Gradient Boosting (XGB) and Support Vector Regression (SVR), can effectively predict surface roughness, with XGB showing the best performance in terms of error metrics such as RMSE and MAPE [31]. Online prediction methods that integrate wavelet transform and convolutional neural networks (WTCNN) enable real-time roughness prediction by capturing dynamic signals, achieving an accuracy rate of 85% [26]. Furthermore, the use of a three-layer backpropagation neural network with real-time sensor data has been shown to optimize prediction accuracy, achieving a mean absolute percentage error of 1.01% (Shang et al., 2024). Machine learning models such as multilayer perceptron regressors have also been effective in predicting roughness in additively manufactured materials, achieving a coefficient of determination greater than 0.95 [27]. Finally, convolutional neural networks have been used to classify surface quality from image data, achieving an accuracy of 82% in predicting surface quality without manual measurement [28].

The combination of these diverse machine learning methodologies improves the assessment and prediction of surface roughness in milling operations, thereby facilitating improved manufacturing quality and efficiency. Furthermore, the various approaches underscore the potential of ML to enhance the precision and efficiency of surface roughness assessment in milling operations, and each approach offers distinctive advantages dependent on the specific application and conditions.

Despite significant advances in the use of machine learning (ML) to predict surface roughness in milling processes, there are gaps in the use of signals from multiple sources to improve prediction accuracy and model robustness. While some studies have integrated multi-sensor data such as vibration, force, and current into ML models, the combined use of these signals with additional inputs such as machine vision or acoustic emissions is still underexplored.

Furthermore, generalizing ML models across domains and signal types remains a challenge, limiting their adaptability to different materials and machining conditions. Although approaches such as physics-informed ML have shown promise in incorporating domain-specific knowledge, their application to multisensory data, particularly in dynamic real-time manufacturing environments, has not been sufficiently addressed.

That is why this article examines the use of machine learning techniques to predict surface roughness in materials processed on a milling machine. Predictions are based on data collected from a variety of sensors installed on the machine, including current transformers, a microphone, and displacement sensors. The article introduces the topic with an overview before proceeding to present a comprehensive account of the research methods and materials employed in Section 2. Section 3 offers a detailed presentation of the results obtained from the machine learning models developed, while Section 4 provides a summary and conclusions. The article concludes with a discussion of the limitations of the study and suggestions for future research directions in Section 5. This study builds on the research previously presented in [32,33].

## 2. Materials and Methods

### 2.1. Research Methodology

**Figure 1:**
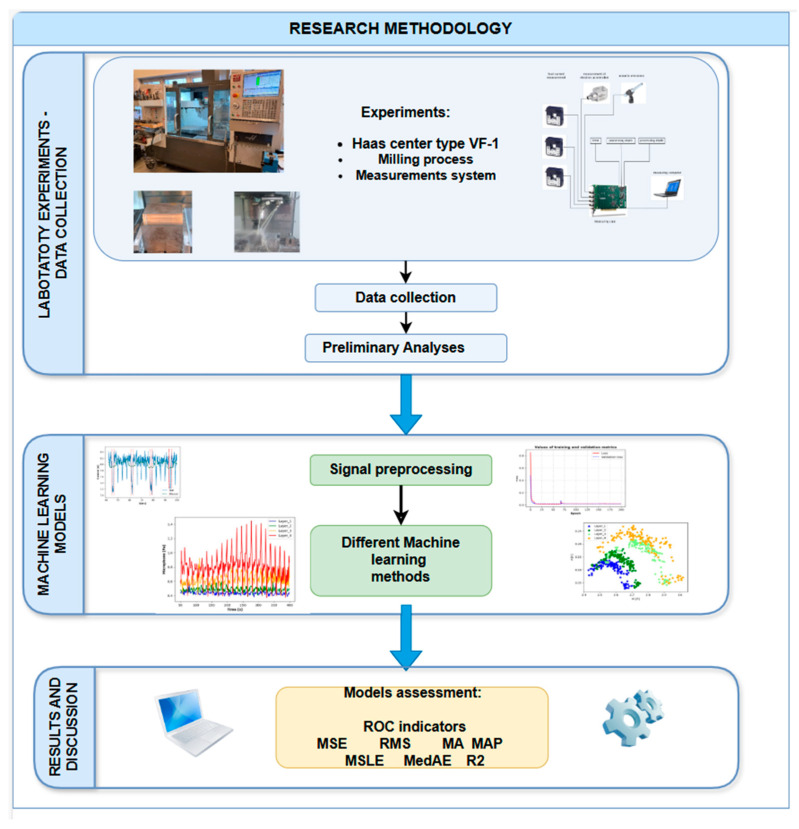
Research Methodology.

The research methodology, as illustrated in Figure 1, contains three principal phases. Laboratory experiments were carried out to collect data, develop machine-learning models, and analyze the results. Each phase comprises a series of defined steps and the use of specific tools with the objective of developing and assessing machine learning models for the recognition of roughness in a milling process.

In the Laboratory Experiments–Data Collection phase, the process is initiated by the configuration and execution of experiments to obtain the requisite data for model training and evaluation. The experiments were carried out using a Haas VF-1 machining center, with a particular focus on the grinding process. Furthermore, the setup comprised a comprehensive measurement system, including current transformers, an accelerometer, and a microphone, as illustrated in the measurement system diagram. During the milling process, data were collected from a variety of sensors, including those that measure vibration, current, and acoustic signals. The aforementioned signals provide indispensable data regarding the machining conditions, which are vital for the prediction of surface roughness. The initial analyses of the collected data were performed with the objective of understanding the nature of the signals and determining any preprocessing requirements. This stage is of great importance, as it allows for the identification of patterns or noise within the raw data, thus ensuring that the data are free from any contamination and suitable for model training.

In the Machine Learning Models phase, the data preprocessing and model development were conducted on the basis of the processed signals. Signals were subjected to pre-processing to eliminate noise and extract pertinent features that could serve to enhance the accuracy of the model. This entailed the use of filtering techniques, in conjunction with potential segmentation, with the objective of focusing on specific periods of direct material processing. This approach ensured that only the most informative data were used for the purposes of model training. Two machine learning models were used for roughness recognition. The first was a multilayer neural network (NN), which was trained to capture nonlinear relationships in the data often present in machining processes. The second was Elastic Net (ELASTINET), a regularization method combining L1 and L2 penalties to handle potential multicollinearity among features and improve the model’s robustness. Furthermore, Elastic Net facilitates the selection of pertinent features, thereby reducing the dimensionality of the predictor set. Models were trained on preprocessed data and metrics such as loss curves were monitored to assess model performance at the end of each epoch. This stage facilitates the optimization of model parameters and the avoidance of overfitting, as evidenced by the training and validation metric graphs.

Subsequently, the performance of the models was evaluated, and the results were interpreted in the Results and Discussion phase. Various performance metrics were calculated with the objective of evaluating the accuracy and reliability of the models in the test set. The metrics include mean squared error (MSE), which measures the average squared difference between actual and predicted roughness values; root mean square error (RMS), which provides an overall measure of prediction error; mean absolute error (MAE), which indicates the average magnitude of prediction errors; and mean absolute percentage error (MAP), which reflects the percentage error in predictions. The metrics used for the assessment of the predictions included the mean squared logarithmic error (MSLE), which focuses on penalizing larger errors more heavily; the median absolute error (MedAE), which assesses the median of absolute prediction errors and provides robustness to outliers; and the coefficient of determination (R2), which indicates how well the model explains the variance in roughness data. The discussion of results comprises an analysis of the aforementioned model assessment metrics, with the objective of determining the relative strengths and weaknesses of each model in roughness prediction. Based on these results, insights are drawn about the effectiveness of neural networks and Elastic Net in predicting roughness, and potential areas for improvement are identified.

### 2.2. Experimental Research

The objective of the investigation was to conduct comprehensive laboratory experiments and construct an extensive measurement database to serve as a basis for the development of advanced methods for signal analysis and parameter identification. The research was carried out on a Haas VF-1 industrial CNC milling machine, which was equipped with an advanced set of measurement sensors, including Hansford three-axis accelerometers with 100 mV/g sensitivity, a microphone with 50 mV/Pa sensitivity and a current transformer sensor with 10 mV/A AC sensitivity. Given the spindle configuration, the vibration sensors were positioned as closely as possible to the spindle bearings once the machine guards were removed. Furthermore, a microphone was placed within the working chamber to facilitate comprehensive audio recording of the acoustic signals produced during the machining process.

The tests were carried out on samples of 1.7225 steel with a hardness of 45 ± 2 HRC (Figure 1), which were machined with two different types of cutters. The cutting tools used were TEFS-E44-CF (Tungaloy Corporation Manufacturer of Metalworking Tools, Iwaki, Fukushima, Japan).

**Figure 2:**
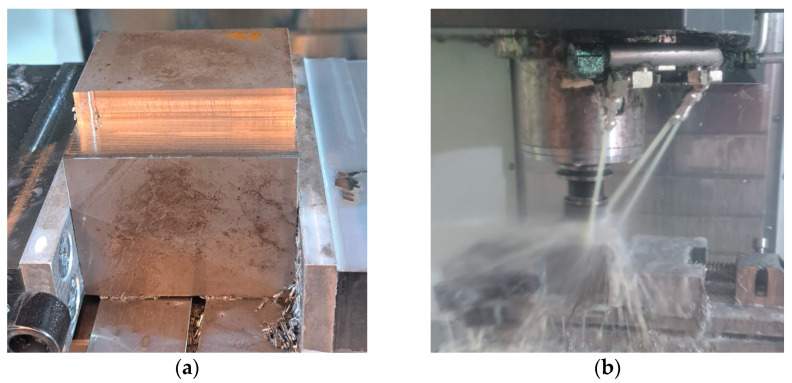
Test sample (**a**) after partial machining and (**b**) during the milling process.

The milling process was conducted with optimized technological parameters, including a milling cutter diameter of 6 mm (four blades), a spindle speed of 5300 rpm, a feed rate of 1270 mm/min, and a cutting speed of approximately 100 m/min. Each sample was machined with a maximum of four layers and following each tool path (Figure 2a,b), the surface roughness (Ra parameter) was precisely measured using a profilometer (TAYLOR HOBSON company, Leicester, UK), with the average value for each path later calculated.

The results from sensors were recorded at a sampling rate of 10 kHz in real time using a sophisticated measurement system comprising signal conditioners, a measurement card, and a laptop with data visualization and recording software.

The experiment involved the acquisition of a comprehensive data set that included a multitude of variables. These included spindle vibration in three directions (x, y, z), noise generated during the process, and machine energy consumption. The data were recorded in six measurement channels.

- Channel A0 records the signals from the *z*-axis sensor, which correspond to the machine’s movement on the *y*-axis.
- Channel A1 records the signals from the *y*-axis sensor, which correspond to the machine movement in the *z*-axis.
- Channel A2 records the signals from the *x*-axis sensor, which correspond to the machine’s movement in the *x*-axis.
- The A3 channel records the noise generated during the process, as captured by the microphone.
- Channel A4 records signals from the spindle current sensor, providing detailed information on the spindle load.
- Channel A5 transmits data from the machine’s current sensor, which provides information on the energy consumption in the milling area.

To guarantee the utmost reliability of the measurements, all sensors and measurement paths were calibrated prior to the start of the test series, thus eliminating the potential for systematic errors. The calibration process involved the execution of tests at dedicated stations and a comparison with reference signals.

**Figure 3:**
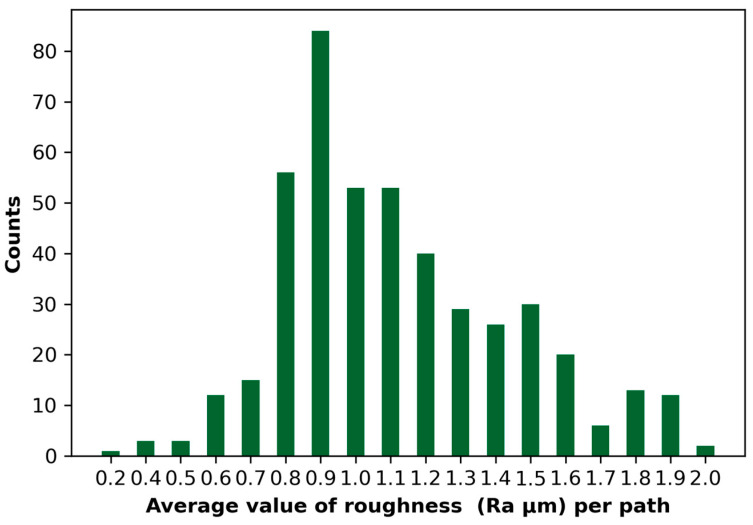
Distribution of the mean surface roughness values (Ra, µm) per path.

Figure 3 shows the distribution of the average surface roughness values (Ra, expressed in µm) obtained for different tool passes during the machining process. Analysis of the results shows that most Ra values are in the range of 0.7–1.0 μm, which means that the process produced surfaces with relatively low and stable roughness in this range. The most common value is 0.9 μm, which forms the dominant center of the distribution. The data distribution is symmetrical with a slight skew towards higher Ra values above 1.2 μm. Extreme roughness values (0.2–0.4 μm and above 1.8 μm) occur very rarely, which can indicate sporadic process instabilities or isolated measurement anomalies.

The dominance of Ra values in the 0.7–1.0 μm range indicates that the machining process has been well-optimized and the technological parameters have minimized surface roughness, ensuring stability and repeatability of the results. However, the appearance of higher Ra values (above 1.2 μm) indicates the need for further analysis of process parameters such as feed rate, depth of cut, or the type of milling cutter used, which may have influenced the increase in roughness. Investigation of these cases could help further optimize the process and reduce higher Ra values.

It can be seen that the process produced surfaces with good quality characteristics, but there is potential for further improvement in stability and precision, particularly by reducing the occurrence of higher roughness values. The results suggest that the machining process was effective, but further studies could further optimize the surface quality achieved.

The data obtained from the experiments were used in the next stage of the investigation, the aim of which was to develop predictive models to assess the quality of the surface expressed by the roughness parameter. Machine learning methods were used for this purpose.

### 2.3. Machine Learning Methods

To develop the prediction models, two machine learning methods were used: Elastic Net (ELASTICNET) and neural networks (NN). Elastic Net combines L1 and L2 regularization, making it effective for feature selection and multicollinear data handling. Neural networks are well-suited for capturing complex, nonlinear relationships due to their multilayered structure, making them highly effective for classification and regression tasks. Together, these methods provide a solid foundation for building accurate and robust predictive models.

Elastic Net is a regression method used in statistics and machine learning that combines two popular techniques: ridge regression and LASSO regression [34,35]. LASSO, which stands for Least Absolute Shrinkage and Selection Operator, is a regression method that uses an L1 regularization technique to penalize the absolute sum of the coefficients. This penalty promotes the selection of relevant variables and can lead to the zeroing of less important variables, effectively selecting a subset of significant variables, which makes LASSO particularly useful for feature selection in high-dimensional data.

The aim of Elastic Net is to address the problems associated with high correlation between explanatory variables and the selection of significant variables. This method uses two types of penalty: the L1 norm (associated with LASSO regression) and the L2 norm (associated with Ridge regression), which increases the stability of the model in the case of collinear variables and reduces their influence through regularization. The Elastic Net model minimizes a cost function that is a combination of these two penalties. This allows it to reduce overfitting by adding penalties to the sum of squared errors and to deal effectively with highly correlated variables by selecting groups of variables rather than individual ones, which is particularly beneficial when variables are interrelated.

Elastic Net offers several key benefits: it allows the selection of significant variables by eliminating unnecessary ones, thereby simplifying the interpretation of results; it ensures model stability, reducing the risk of overfitting; and it handles collinearity better than either LASSO or Ridge methods alone. Practical applications of Elastic Net include situations where the data contain many correlated variables. Elastic Net is particularly useful when standard linear regression or individual LASSO and ridge methods prove inadequate for dealing with collinearity and reducing the number of variables.

In linear models [36,37], we consider the dependence:

(1) $$y_{i}=\alpha_{0}+\alpha_{1}x_{i1}+\ldots +\alpha_{m}x_{im}+\varepsilon_{i}\;$$

We define

(2) $$Y=\left[\begin{array}{c} y_{1}\\ y_{2}\\ \vdots \\ y_{n} \end{array}\right],\;X=\left[\begin{array}{ccccc} 1 & x_{11} & x_{12} & \ldots & x_{1m}\\ 1 & x_{11} & x_{12} & \ldots & x_{1m}\\ \vdots & \vdots & \vdots & \vdots & \vdots \\ 1 & x_{n1} & x_{n2} & \ldots & x_{nm} \end{array}\right]=\left[\begin{array}{c} x_{\left(1\right)}\\ x_{\left(2\right)}\\ \vdots \\ x_{\left(n\right)} \end{array}\right]\;\theta =\left[\begin{array}{c} \theta_{0}\\ \theta_{1}\\ \vdots \\ \theta_{m} \end{array}\right],\;\varepsilon =\left[\begin{array}{c} \varepsilon_{1}\\ \varepsilon_{2}\\ \vdots \\ \varepsilon_{n} \end{array}\right],$$

From Equation (2) we obtain

(3) Y=Xθ+ε 

to estimate the parameters *θ* we use the least squares method [37,38]. For this purpose, we solve the following task

(4) $$\underset{\theta }{min}{||Y-X\theta ||}^{2}$$

where ${||Y-X\theta ||}^{2}=\sum_{1}^{n}{\left(f\left(y_{i}\right)-<x_{\left(i\right)},\theta >\right)}^{2}$, and <.,.> denotes the dot product.

According to the Gauss–Markov theorem, if $det\left(X^{T}X\right)\neq 0$, then the best estimator in the mean square sense is defined by the following formula:

(5) $$\hat{\theta }={\left(X^{T}X\right)}^{-1}X^{T}Y$$

The structural parameter estimator $\hat{\theta }$ is the Best Linear Unbiased Estimator [36].

In the case where the input variables are collinear, unbiased estimates have a larger variance, and the prediction of the model is less accurate. However, in some situations, biased estimates have a smaller variance and smaller error mean square than unbiased estimators. To reduce overdimensionality, Elastic Net [39] was used. The parameters of the linear model (1) are determined by solving the following task:

(6) $$\underset{\theta }{min}\left\{\sum_{i=1}^{n}{\left(y_{i}-<x_{\left(i\right)},\theta >\right)}^{2}+\lambda P_{\alpha }\left(\theta \right)\right\},$$

where λ>0, 0≤α≤1, and $P_{\alpha }$ denotes the penalty given by the following formula:

(7) $$P_{\alpha }\left(\theta \right)=\alpha {∥\theta ∥}_{L_{1}}+\frac{1-\alpha }{2}{∥\theta ∥}_{L_{2}}=\sum_{j=1}^{m}\left(\alpha \left|\theta_{j}\right|+\frac{1-\alpha }{2}\theta_{j}^{2}\right).$$

The penalty $P_{\alpha }\left(\theta \right)$ is a linear combination of the norms of the vector of estimators θ in the spaces $L_{1}$ and $L_{2}$. For *α* = 0, we have the ridge regression, while for *α* = 1, we have LASSO [36,39]. We define the predicted values as ${\hat{y}}_{i}=<x_{\left(i\right)},\hat{\theta }>$ and 1≤i≤n.

Neural networks (NN) are one of the most widely used types of machine learning algorithms. A Neural network is usually utilized for classification or regression analysis [38,39,40]. The structure of a neural network consists of inputs, weights (parameters), aggregation functions, activation functions, and outputs. When we apply the multilayer networks, then the inputs of the first layer are the predictors from the training set, but the inputs of the next layers are defined as outputs of the activation function of the current layer. In neural networks, the aggregation function is defined as a linear combination of the inputs and weights and the result of the aggregation function is sent to the activation function $g_{j}$, which is defined as follows:

(8) $$Z_{j}\left(x\right)=g_{j}\left(w_{j}Z_{j-1}\left(x\right)+\alpha_{j}\right)$$

where $w_{j}$ denotes the weights, $\alpha_{j}$ for j=1,2,…,K denotes the bias, and for input layer and we put $Z_{0}\left(x\right)=x$. The results of the activation function from the last (output) layer are defined by a result obtained from the neural network. Thus, from (7), the last layer is defined as follows:

(9) $$Z_{K}\left(x\right)=g_{K}\left(w_{K}g_{K}\left(w_{K-1}Z_{K-2}\left(x\right)+\alpha_{K-1}\right)+\alpha_{K}\right)=\ldots \;\;\;\;\;\;\;\;\;\;\;\;\;\;\;\;\;=g_{K}\left(w_{k}g_{K-1}\left(w_{K-1}g_{K-2}\left(\ldots g_{2}\left(w_{2}g_{1}\left(w_{1}x+\alpha_{1}\right)+\alpha_{2}\right)\ldots \right)+\alpha_{K-1}\right)+\alpha_{K}\right)$$

The ReLu activation function was used to analyze the roughness of the processed material, which is defined as follows:

(10) $$\mathrm{ReLu}\left(s\right)=max\left\{0,s\right\}$$

For the regression problem based on neural network, the task consists of solving the problem:

(11) $$min\;R\left(\{w_{j}{\}}_{1\leq j\leq K},\{\alpha_{j}{\}}_{1\leq j\leq K}\right).$$

where the objective function is defined as follows:

(12) $$R\left(\{w_{j}{\}}_{1\leq j\leq K},\{\alpha_{j}{\}}_{1\leq j\leq K}\right)=\frac{1}{n}\sum_{i=1}^{n}{\left(y_{i}-Z_{K}\left(x_{i}\right)\right)}^{2}$$

denotes mean squared error and output layer $Z_{k}\left(\cdot \right)$ is defined by formula (9). To solve problem (10), we usually apply the backward propagation algorithm.

### 2.4. Developed Models Evaluation

To assess the quality of the developed models, the test set $D_{test}=\left\{\left(x_{\left(i\right)},y_{i}\right):x_{\left(i\right)}\in R^{m},y_{i}\in R,1\leq i\leq k\right\}$ was used. For the obtained model after the training process, the predicted values of the dependent variable ${\hat{y}}_{i}$ corresponding to the signal readings $x_{\left(i\right)}\in R^{m}$, 1≤i≤k were determined, thus obtaining the sequence ${\left\{{\hat{y}}_{i}\right\}}_{1\leq i\leq k}$. The evaluation of roughness recognition based on this model is carried out based on the following metrics [36,37,38,39,41].

The coefficient of determination shows what part of the variability of the dependent variable is explained by the model and is estimated as follows:

(13) $$R^{2}=1-\frac{\sum_{i=1}^{k}{\left(y_{i}-{\hat{y}}_{i}\right)}^{2}}{\sum_{i=1}^{k}{\left(y_{i}-\bar{y}\right)}^{2}},$$

where $\bar{y}=\frac{1}{k}\sum_{i=1}^{k}y_{i}$.

Mean absolute error (MAE) denotes the expected value of the absolute differences between actual and predicted values in $L_{1}$ norm. The MAE metric is calculated as follows:

(14) $$MAE=\frac{1}{k}\sum_{i=1}^{k}|y_{i}-{\hat{y}}_{i}|$$

Mean absolute percentage error (MAPE) denotes the mean absolute error in relation to the absolute actual value and is calculated as follows:

(15) $$MAPE=\frac{1}{k}\sum_{i=1}^{k}\frac{\left|y_{i}-{\hat{y}}_{i}\right|}{max\left(\epsilon ,\left|y_{i}\right|\right)}$$

where ϵ>0 is an arbitrarily small positive value to avoid undefined results when y is zero.

Mean squared error (MSE) denotes the expected value of the squared error (squared differences between actual and predicted values) and is estimated as follows:

(16) $$MSE=\frac{1}{k}\sum_{i=1}^{k}{\left(y_{i}-{\hat{y}}_{i}\right)}^{2}$$

Root mean squared error (RMSE) is an average expected difference between actual and predicted values and is estimated as follows:

(17) $$RMSE=\sqrt{\frac{1}{k}\sum_{i=1}^{k}{\left(y_{i}-{\hat{y}}_{i}\right)}^{2}}$$

Median absolute error (MedAE) is a median of a sequence of absolute errors (differences between actual and predicted values) and is estimated as follows:

(18) $$MedAE=median\left(\left|y_{1}-{\hat{y}}_{1}|,|y_{2}-{\hat{y}}_{2}|,\ldots ,|y_{k}-{\hat{y}}_{k}\right|\right)$$

Mean squared logarithmic error (MSLE) denotes the expected value of the squared logarithmic error (squared logarithm of the quotient of actual and predicted values shifted by 1) and is estimated as follows:

(19) $$MSLE=\frac{1}{k}\sum_{i=1}^{k}{\left(log\frac{1+y_{i}}{1+{\hat{y}}_{i}}\right)}^{2}$$

## 3. Results and Discussion

### 3.1. Processing of Sensor Signals

To assess the vibration and acoustic characteristics of material processing, the absolute values from both the accelerometer and microphone readings were analyzed for the collected signals. To ensure accurate timing across all sensors, the readings were carefully synchronized with data from the current transformer. Furthermore, instances of direct material processing by the milling cutter were identified. Only the data segments corresponding to these direct processing periods were selected for further analysis, as they provide the most relevant information regarding tool–material interactions. Any readings taken when the milling cutter was outside the material were excluded as they do not contribute to insights into the machining process. This focused selection of data segments allows for the isolation of the signals that are most indicative of actual milling conditions, thereby facilitating a more precise and reliable analysis. To automate the determination of moments related to direct material processing, the time series obtained from the current transducer, ${\left\{\xi_{t}\right\}}_{1\leq t\leq n}$, is filtered using the Savitzky–Golay filter:

(20) $${\bar{\xi }}_{j}=\sum_{i=\frac{1-m}{2}}^{\frac{m-1}{2}}c_{i}\xi_{j+i}$$

where for $\frac{m+1}{2}\leq j\leq n-\frac{m+1}{2}$ quantities $c_{j}$ denote convolution coefficients. Then, for the series ${\left\{{\bar{\xi }}_{t}\right\}}_{\frac{m+1}{2}\leq t\leq n-\frac{m+1}{2}}$, drops were determined, e.g., current (holes), moments where the cutter was outside the processed material, thus obtaining the sequence ${\left\{n_{i}\right\}}_{1\leq i\leq s}$, where $n_{s}\leq n$.

**Figure 4:**
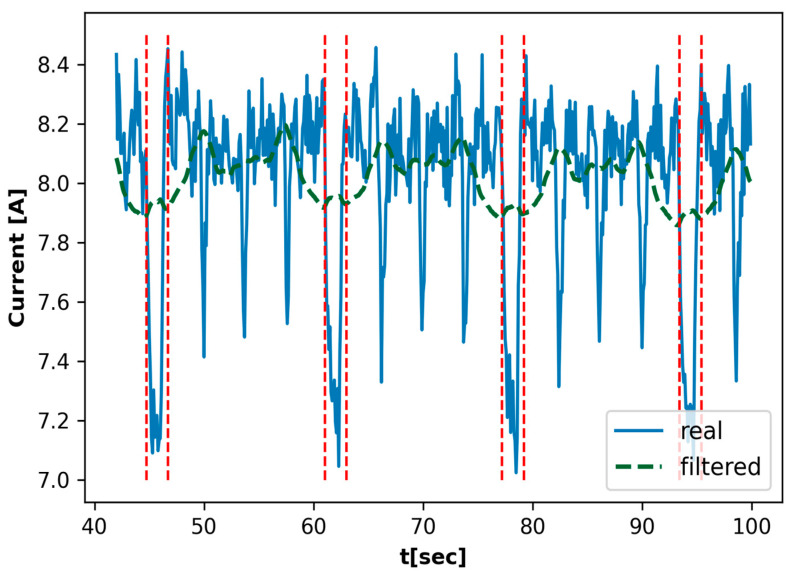
Filtered current signal with nonprocessing intervals marked.

Figure 4 illustrates the filtering of the signal received from the current transducer, thus identifying the instances where the material was not subjected to processing.

Figure 4 illustrates the distinction between the actual current readings, represented by the blue line, and the filtered signal, represented by the green dashed line. This filtered signal was obtained by applying the Savitzky–Golay filter. The application of this filter serves to mitigate the impact of fluctuations in the current signal, thereby facilitating a more discernible examination of the underlying patterns. The red dashed vertical lines serve to highlight specific intervals where a reduction in current is observed, indicating instances when the milling cutter is not in direct contact with the material. These periods correspond to instances where the cutter is not in direct contact with the material, resulting in a temporary reduction in current due to the absence of cutting resistance. By identifying these nonprocessing intervals, the analysis focuses on the segments where actual tool–material interaction occurs. This filtered approach helps to isolate the relevant segments of the data associated with direct processing, removing unnecessary noise from intervals without contact. The result is a more refined dataset that is better suited for analyzing vibration and acoustic characteristics related to milling operations.

After the filtering, the sequences ${\left\{\xi_{t}\right\}}_{n_{k}\leq t\leq n_{k+1}}$ were processed, for which the material was directly processed from the moment $n_{k}$ to the moment $n_{k+1}$, where k<s. In many cases, the series contain hidden factors [30,31], so it is important to extract information from the analyzed series. In the case under consideration, ${\left\{\xi_{t}\right\}}_{n_{k}\leq t\leq n_{k+1}},$ a sequence of quantiles was determined for each sequence ${\left\{q_{p}\right\}}_{p\in \{0,0.05,0.1,\ldots ,1\}}\in R^{21}$, where the quantile of order p is determined as follows:

(21) $$q_{p}=min\left\{\xi_{\left(i\right)}:i\geq \left[p\left(n_{k+1}-n_{k}\right)\right]\right\}$$

whereas ${\left\{\xi_{\left(i\right)}\right\}}_{1\leq i\leq n_{k+1}-n_{k}}$ is an ordered sequence formed from the sequence ${\left\{\xi_{t}\right\}}_{n_{k}\leq t\leq n_{k+1}}$, whereas $\left[.\right]$ means rounding up. Determining quantiles gives us information about the shape of the empirical distribution [30,31]. For each cutter path, the average roughness was estimated.

### 3.2. Investigation of the Dependence of Roughness on the Signals from Sensors

To evaluate the surface roughness of the machined material, the signals collected from the multiple sensors were subjected to analysis and processing. These signals were segmented on the basis of the moments of direct processing, thus facilitating a concentrated examination of the tool–material interactions that are pertinent to the prediction of surface quality.

For each of the signals, ${\left\{\xi_{t}\right\}}_{n_{k}\leq t\leq n_{k+1}}$ empirical distributions and the corresponding sequence of quantiles ${\left\{q_{p}\right\}}_{p\in \{0,0.05,0.1,\ldots ,1\}}\in R^{21}$ were determined.

Let $D=\left\{\left(x_{i},y_{i}\right):x_{i}\in R^{m},y_{i}>0,1\leq i\leq n\right\}$ denote the training data set. The task was to recognize the roughness of the machined material $y_{i}$ from the value of the sequence of realizations $x_{i}\in R^{m}$ obtained after preprocessing the signals ${\left\{\xi_{t}^{i}\right\}}_{n_{k}\leq t\leq n_{k+1}}$ for k=1,2,…,s−1 from sensors i∈{A0,A1,A2,A3,A4,A5}.

For this purpose, Elastic Net (to reduce the overdimensionality of the predictors) [31,32,33] and a multilayer neural network were used to determine the dependencies.

**Figure 5:**
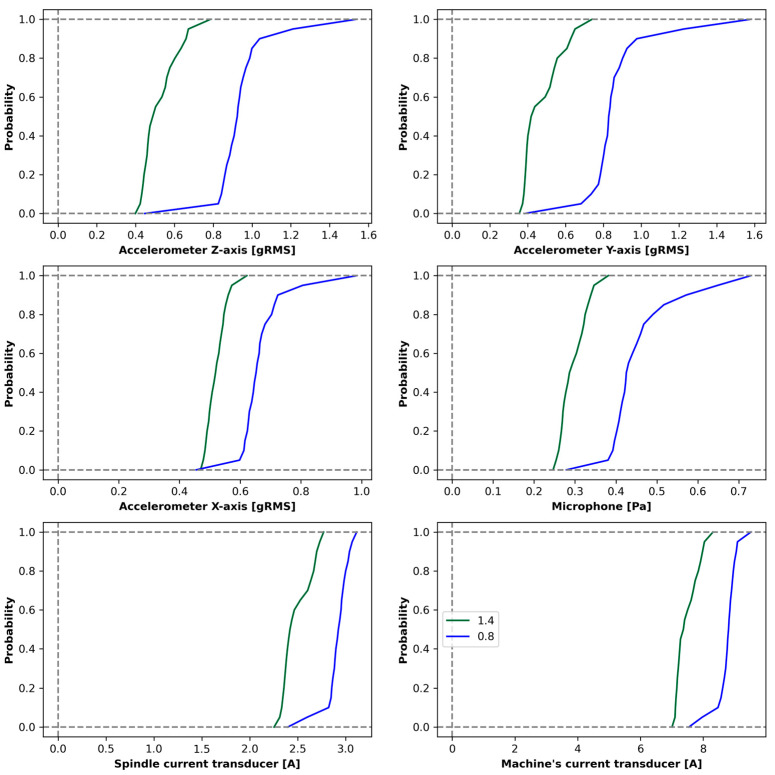
Empirical distributions of signals along the processing path with corresponding roughness levels.

Figure 5 presents an example of the empirical distributions of signals along a processing path and their corresponding roughness values. This visualization allows an examination of the relationship between signal characteristics and the surface roughness of the machined material.

For each run, the order $p\in \left\{0,0.05,0.1,\ldots ,1\right\}$ quantiles (21 quantiles, where the order quantile 0 corresponds to the smallest value of the reading sequence, while the order quantile 1 corresponds to the largest value) and the average roughness from the milling path. In this way, a data set was created: $D=\left\{\left(x_{i},y_{i}\right):x_{i}\in R^{126},y_{i}\in R_{+},1\leq i\leq n\right\}$, where n− the number of analyzed cases. The figure shows sample distributions for each of the sensor signals and the roughness value.

The entire set of 458 cases was divided into a training set that contained 80% of the cases and a test set that contained 20% of the cases. The dependence of roughness on the quantile values of the signals read from the sensors was identified in the training set. The regularization parameter was selected for Elastic Net *λ* = 0.001 and the LASSO influence coefficient and the ridge regression *α* = 0.25. For values λ and α, the highest value of the determination coefficient was obtained.

In the case of a neural network, the model for the dependence of roughness on the quantile values of the sensor signals. Table 1 describes the architecture of a neural network model used to predict material roughness based on the quantile values of sensor signals. The network consists of an input layer with 126 features, followed by three dense (fully connected) layers with decreasing output sizes: 80, 50, and 10 neurons, each using the ReLu activation function. The final output layer has a single neuron with ReLu activation, representing the predicted roughness. The model has a total of 14,731 trainable parameters, allowing it to capture complex relationships between signal quantiles and surface roughness. The number of training epochs is 200. Validation was used in the set containing a 15% batch.

The MSE indicator was selected as the primary metric for assessing the quality of training for the neural network in the regression model. The MSE serves as a function to measure the efficacy of the model in predicting target values by calculating the average squared difference between the predicted and actual values. A lower MSE indicates a better fit, reflecting smaller discrepancies between the model predictions and the observed data. Furthermore, the MSE was employed as the validation metric, providing a consistent measure of model performance in both the training and validation phases, facilitating effective monitoring of model accuracy and potential overfitting.

**Figure 6:**
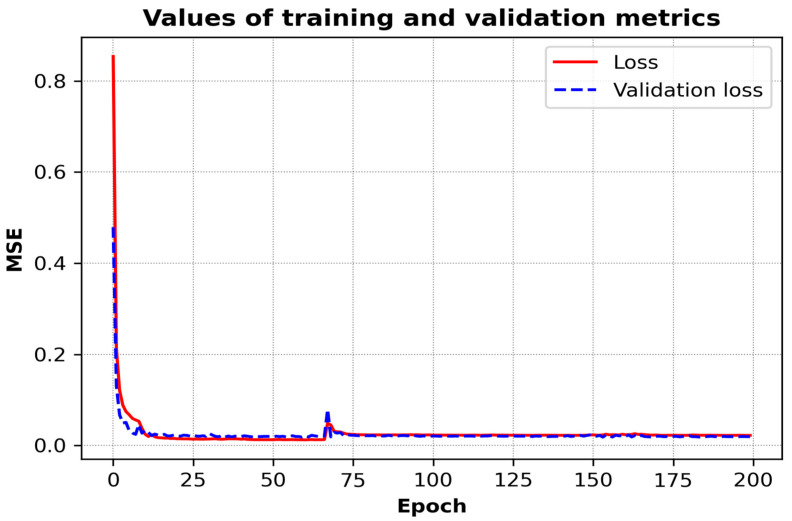
The process of training a neural network.

Figure 6 presents a visual representation of the training process of a neural network, showcasing the values of the mean squared error (MSE) metric for both the training and validation sets over the course of 200 epochs. The red line represents the training loss (MSE on the training data), while the blue dashed line represents the validation loss (MSE on the validation data).

In the initial stages of the training process, both the training and the validation MSE are relatively high, indicating a poor fit. However, as training progresses, the MSE values rapidly decrease, demonstrating that the model is learning and improving its predictions. Following approximately 25 epochs, the loss stabilizes and converges to a low value for both the training and validation sets, indicating that the model has reached a state of minimal error and is well generalized. After approximately 25 epochs, the MSE values for both the training and validation sets fall below 0.05. This indicates that the preprocessing approach effectively extracts meaningful features from the data. In particular, the use of the Empirical Cumulative Distribution Function (ECDF) for each signal proves to be highly informative, capturing key details related to the quality of the material machining process.

The rapid convergence of the neural network highlights the robustness of the pre-processing strategy, which ensures that the data fed into the model are well-structured and contain minimal noise. Furthermore, the low MSE values suggest that the ECDF-based features provide a comprehensive representation of the machining conditions, allowing the model to generalize effectively across the test cases. This highlights the importance of pre-processing, not only to reduce the complexity of the raw signals but also to improve the predictive accuracy of advanced machine learning models in assessing surface quality. There is no significant divergence between the two curves, which suggests that overfitting has been successfully avoided and that the model performs consistently across both the training and validation datasets.

The validation of each developed model was conducted using a separate test set with the objective of evaluating its ability to generalize beyond the training and validation data. Figure 5 and Figure 6 illustrate the performance of the models in predicting surface roughness, showing both predicted roughness and actual roughness measurements along the milling path. By comparing these curves, it is possible to assess the accuracy and consistency of the recognition of roughness across various segments of the path.

**Figure 7:**
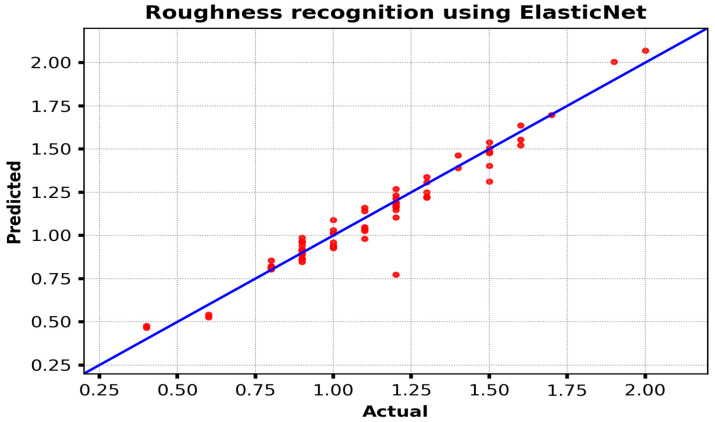
The roughness recognition using Elastic Net.

Figure 7 illustrates the performance of the Elastic Net model in predicting surface roughness, with actual roughness values (*x*-axis) compared to predicted values (*y*-axis) on the test set. The red dots represent the individual roughness predictions for a given instance. The blue line represents the ideal-fit line, indicating a perfect correlation between the predicted and actual values. The proximity of the red dots to the best-fit line indicates the degree of alignment between the model’s predictions and the actual measurements. Most of the data points are situated in proximity to the line, indicating that the model predictions are, for the most part, accurate and within a reasonable margin of error.

Nevertheless, there are some discernible divergences from the ideal line, with a few points situated either above or below it. These deviations indicate instances where the Elastic Net model slightly overestimated or underestimated the actual roughness values. Although the model captures the general trends effectively, these discrepancies may reflect limitations in the model’s capacity to capture certain variations in the input data or complex factors influencing roughness. Overall, this plot highlights the model’s effectiveness in predicting roughness while also pointing to potential areas for improvement, such as fine-tuning model parameters or incorporating additional data to enhance prediction accuracy.

**Figure 8:**
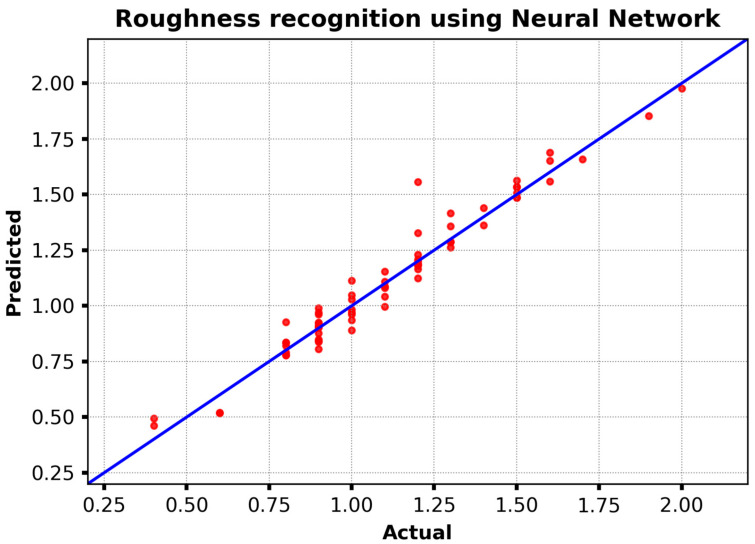
The roughness recognition using neural network.

Figure 8 illustrates the performance of a neural network model in predicting surface roughness, with actual roughness values compared to predicted values on the test set. Each data point represents a single prediction for a given instance, and the diagonal line represents the ideal fit, where the predicted and actual values would be perfectly fitted.

The points are situated in close proximity to the ideal line, which suggests that the neural network model effectively captures the relationship between the input features and roughness measurements. However, some points deviate from this line, indicating instances where the model overestimated or underestimated the roughness. These deviations suggest potential areas for improvement in the model’s accuracy, potentially due to the presence of complex patterns in the data that may not have been fully captured. Overall, this visualization demonstrates the neural network’s ability to predict roughness accurately while highlighting opportunities for further refinement.

Table 2 presents a comprehensive comparison of performance metrics for roughness recognition on the test set, contrasting the Elastic Net and neural network (NN) models. Key metrics (Equations (11)–(17)) provide a comprehensive overview of the accuracy, consistency, and overall fit of each model to the test data.

The NN model shows better performance compared to the Elastic Net model across most of the metrics evaluated. In particular, the model exhibits a lower mean squared error (MSE) of 0.00483 compared to the Elastic Net model, which displays an MSE of 0.00587. This indicates that the average squared difference between the predicted and actual values is smaller for the NN model. This pattern is further substantiated by the root mean square (RMS) error, which is 0.06952 for the NN model and 0.07658 for the Elastic Net model. This also indicates that the NN model exhibits smaller overall errors. Similarly, the mean absolute error (MAE) and median absolute error (MedAE) values for the NN model are lower than those for the Elastic Net model, indicating that the NN model not only has a lower average error but also fewer large deviations from actual values.

The mean absolute percentage error (MAPE) values are comparable between the two models, with the neural network (NN) model exhibiting a slight advantage (0.04977 vs. 0.05008). This indicates that the two models have comparable accuracy in terms of percentage-based error. The MSLE for the NN model (0.00110) is also lower than for Elastic Net (0.00141), indicating that the NN model is slightly more effective at minimizing errors on a logarithmic scale. This can be particularly relevant for distributions with outliers or skewed data.

The R² values, which quantify the extent to which each model explains the variance in the roughness data, are both high, indicating robust predictive power. However, the NN model achieves a higher R² of 0.95060 compared to 0.94005 for Elastic Net, indicating a better overall fit to the test data and a stronger ability to capture the underlying relationships.

The NN model demonstrates superior performance in predicting surface roughness across most of the metrics evaluated compared to the Elastic net model. The lower error values and the higher R² values indicate that the neural network model is more effective in capturing the complexities of the data, making it a preferable choice for roughness recognition tasks in this set of tests. The neural network model’s advantage across these metrics suggests its potential robustness and suitability for applications requiring high accuracy in roughness prediction.

An additional analysis was performed to compare the differences in prediction between the Elastic Net and neural network models. The Wilcoxon signed-rank test was used to assess the significance of these differences. The null hypothesis for this test is that the distribution of the differences between the predictions of the two models is symmetric around zero, implying that the median of the prediction differences is zero. The calculated test statistic was 1190.0, with a corresponding p-value of 0.9166. Given the high p-value, we cannot reject the null hypothesis. This indicates that there is no statistically significant difference between the predictions made by the Elastic Net and neural network models.

This result suggests that, despite differences in model structure and computational complexity, the predictions of both models are comparable in terms of their overall distribution, reinforcing their validity as tools for predicting surface roughness. However, further analysis may focus on specific scenarios or subsets of the data to explore potential nuanced differences between developed models.

## 4. Conclusions

The main objective of the study conducted was to explore the potential of machine learning techniques, specifically Elastic Net and neural networks, to predict surface roughness in milling processes using data from multiple sensors. Integration of signals from vibration, acoustic, and current sensors yielded a comprehensive data set that allowed a detailed analysis of tool–material interactions during machining. This approach highlighted the importance of signal preprocessing, including the use of the Savitzky–Golay filter, which effectively isolated pertinent machining signals by eliminating noise and identifying instances of direct tool–material contact. The aforementioned preprocessing steps ensured the reliability and accuracy of the data used for the purposes of model training and evaluation.

The performance of the two predictive models, namely Elastic Net and neural networks, was subjected to a comprehensive evaluation. The two models demonstrated high accuracy in predicting surface roughness, with the neural network outperforming Elastic Net in most of the evaluation metrics. The neural network exhibited superior predictive capabilities, with an R² value of 0.95, compared to 0.94 for Elastic Net. Additional metrics, such as mean squared error (MSE) and root mean square error (RMSE), provided further validation of the neural network’s capacity to capture complex, nonlinear relationships within the data set. In contrast, the Elastic Net model demonstrated effective handling of multicollinearity and performed feature selection to reduce dimensionality.

The experimental results demonstrated that the milling process yielded surfaces with low and consistent roughness values, predominantly within the range of 0.7–1.0 μm, indicative of a highly optimized machining process. However, occasionally, higher roughness values (exceeding 1.2 μm) were also recorded. These discrepancies indicate the potential influence of factors such as feed rate, cutting speed, and tool condition, which could be subjected to further analysis and optimization in order to enhance the process’s stability and precision.

The study demonstrates the methodological advancements achieved through the integration of machine learning and multi-sensor data. The Elastic Net method allowed the simplification of the set of predictors and the resolution of collinearity problems. On the contrary, neural networks demonstrated superior performance in modeling the intricate dependencies between sensor data and surface roughness. Together, these approaches demonstrated the potential to leverage machine learning to improve the prediction and control of machining outcomes.

From a practical perspective, this research demonstrated the feasibility of using advanced machine learning models to predict surface roughness accurately, enabling better process control and reducing the need for secondary finishing operations. Integration of sensor data and predictive modeling offers significant benefits for manufacturing efficiency and quality assurance, making it a valuable tool for smart manufacturing systems.

## 5. Limitations and Future Research

Although the study achieved encouraging outcomes in forecasting surface roughness through machine learning and multisensory data analysis, it is constrained by several factors that offer directions for future research. One of the primary constraints is the scope of the data used. The research was carried out on a specific material (1.7225 steel) and a defined set of machining parameters, which limits the generalizability of the findings. To enhance the generalizability of the models, future studies should validate their performance across a more diverse range of materials, tool configurations, and operational conditions.

The second limitation pertains to the integration of sensor data. Although the study incorporated vibration, acoustic, and current signals, other valuable data sources, such as machine vision or advanced acoustic emission techniques, were not used. The incorporation of these additional modalities in future research could prove beneficial in enriching the datasets, thereby enhancing the models’ predictive accuracy and robustness.

Additionally, the study’s emphasis on offline prediction of surface roughness demonstrates the necessity for real-time predictive models capable of functioning in dynamic manufacturing environments. The implementation of real-time systems would provide the ability to make immediate adjustments to the machining parameters, ensuring consistent surface quality throughout the production process.

The interpretability of machine learning models, particularly those based on neural networks, represents another challenge. Despite the high accuracy demonstrated by these models, their black-box nature constrains the actionable insights they can provide into the relationships between machining parameters and surface roughness. It is recommended that future research should aim to develop more interpretable models, which could offer a deeper understanding and greater trust in their predictions.

Furthermore, the current approach relies fully on machine learning methods to capture data patterns without integrating domain-specific knowledge. The integration of physics-informed machine learning methodologies could enhance the precision and resilience of the predictions, particularly in scenarios characterized by limited data or substantial variability in machining conditions.

Furthermore, the study identified occasional anomalies, such as higher roughness values, which were likely caused by unexpected variations in machining conditions or noise in the data. Future research should concentrate on the implementation of sophisticated anomaly detection techniques in order to address such outliers and enhance the stability of the predictions.

As previously stated, future research can build on the findings of this study to create more generalized, efficient, and interpretable models for surface roughness prediction. Such advancements will not only promote the adoption of machine learning in smart manufacturing but also support the development of more precise, adaptable, and sustainable production processes across various industries.

## Figures and Tables

**Table 1:** Neural network model for predicting roughness based on quantile values of sensor signals.

Layer (Type)	Output Shape	Parameters	Activation
Input Layer	(1, 126)	0	-
Dens 1	(1, 80)	10160	ReLu
Dens 2	(1, 50)	4050	ReLu
Dens 3	(1, 10)	510	ReLu
Output Dense	(1, 1)	11	ReLu

**Table 2:** Key metrics for roughness recognition on the test set for Elastic Net and neural network.

	MSE	RMS	MAE	MAP	MSLE	MedAE	R^2^
Elastic Net	0.00587	0.07658	0.05185	0.05008	0.00141	0.04196	0.94005
NN	0.00483	0.06952	0.04852	0.04977	0.00110	0.03539	0.95060

## Data Availability

The data presented in this study are available on request from the corresponding author.
